# Extracellular Vesicles in Diagnosing Chronic Coronary Syndromes the Bumpy Road to Clinical Implementation

**DOI:** 10.3390/ijms21239128

**Published:** 2020-11-30

**Authors:** Mirthe Dekker, Farahnaz Waissi, Nathalie Timmerman, Max J. M. Silvis, Leo Timmers, Dominique P. V. de Kleijn

**Affiliations:** 1 Department of Vascular Surgery, University Medical Centre Utrecht, Heidelberglaan 100, 3584 CX Utrecht, The Netherlands; m.dekker-17@umcutrecht.nl (M.D.); f.waissi-2@umcutrecht.nl (F.W.); N.Timmerman-2@umcutrecht.nl (N.T.); 2 Department of Cardiology, Amsterdam University Medical Centre, Mijbergdreef 9, 1105AZ Amsterdam, The Netherlands; 3 Department of Cardiology, University Medical Centre Utrecht, 3584 CX Utrecht, The Netherlands; M.J.M.Silvis@umcutrecht.nl; 4 Department of Cardiology, St. Antonius Hospital Nieuwegein, 3435 CM Nieuwegein, The Netherlands; l.timmers@antoniusziekenhuis.nl; 5 Netherlands Heart Institute, 3511 EP Utrecht, The Netherlands

**Keywords:** chronic coronary syndrome (CCS), coronary artery disease (CAD), angina pectoris, extracellular vesicles (EVs), biomarker, protein, liquid biopsy cardiovascular disease

## Abstract

Coronary artery disease (CAD), comprising both acute coronary syndromes (ACS) and chronic coronary syndromes (CCS), remains one of the most important killers throughout the entire world. ACS is often quickly diagnosed by either deviation on an electrocardiogram or elevated levels of troponin, but CCS appears to be more complicated. The most used noninvasive strategies to diagnose CCS are coronary computed tomography and perfusion imaging. Although both show reasonable accuracy (80–90%), these modalities are becoming more and more subject of debate due to costs, radiation and increasing inappropriate use in low-risk patients. A reliable, blood-based biomarker is not available for CCS but would be of great clinical importance. Extracellular vesicles (EVs) are lipid-bilayer membrane vesicles containing bioactive contents e.g., proteins, lipids and nucleic acids. EVs are often referred to as the “liquid biopsy” since their contents reflect changes in the condition of the cell they originate from. Although EVs are studied extensively for their role as biomarkers in the cardiovascular field during the last decade, they are still not incorporated into clinical practice in this field. This review provides an overview on EV biomarkers in CCS and discusses the clinical and technological aspects important for successful clinical application of EVs.

## 1. Introduction

Coronary artery disease (CAD) remains one of the most important killers among the entire world, despite tremendous improvements in diagnostic and therapeutic strategies [1]. CAD comprises acute coronary syndromes (ACS) and chronic coronary syndromes (CCS, e.g., stable angina). The underlying pathophysiology that causes CAD is known as atherosclerosis [1]. This is a longstanding, continuous process of accumulation and progression of plaque material within the vessel wall [2]. Atherosclerotic plaques are often stable for long periods and can eventually cause a diminished oxygen supply to the heart muscle during exertion. This causes ischemia and subsequent chest pain [3]. The resulting clinical syndrome is known as CCS, for which medical or interventional therapies are generally required. Plaque rupture or plaque erosion initiates an acute thrombotic luminal occlusion that can cause acute blockage of one the coronary vessels, resulting in ACS and, subsequently, myocardial infarction [4]. ACS requires immediate revascularization of the affected vessel.

**Figure 1:**
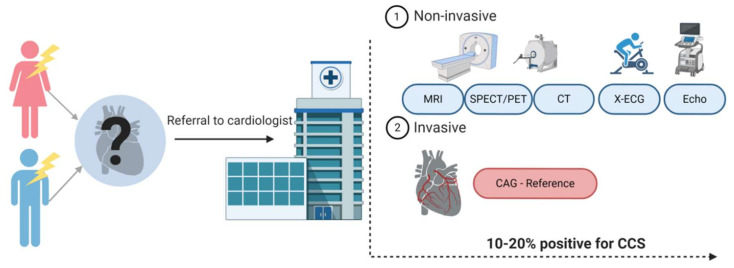
Diagnostic track of patients with chest pain suspected for a chronic coronary syndrome (CCS). Patients suspected of CCS are often referred to a cardiologist. Patients undergo either noninvasive or invasive tests. The choice for one of the tests is based on the pre-test probability of a patient having CCS and availability in the hospital. Created with BioRender.com.

ACS is often quickly diagnosed with either an abnormal electrocardiogram (ECG) or elevated cardiac biomarkers, such as high-sensitive cardiac troponin (hs-cTn), indicating cell damage of the myocardium. Diagnosing CCS appears to be more complicated. Figure 1 provides an overview of the diagnostic workflow of a patient presenting with chest pain at the general practitioner and the diagnostic possibilities once referred to the cardiologist. The reference standard for CCS is still coronary angiography (CAG), but considering its invasive character, this is used with caution [1,5]. Currently, the most used noninvasive strategies are coronary computed tomography (CT) or myocardial perfusion imaging (MPI). The diagnostic accuracy of these different imaging modalities is relatively high (80–90%), but only 10–20% of symptomatic patients turn out to have CCS [6]. The low number of patients suffering from the actual disease are the result of an increasing use of these test modalities in the low-risk population [7,8,9]. They are becoming more and more subject of debate because of unnecessary radiation exposure for the patient and high costs. A reliable, blood-based biomarker would therefore be important to improve the diagnostic strategy around patients suspected for CCS. Until now, no such biomarker exists.

Since the early 1960s, there is a growing interest for extracellular vesicles (EVs) as potential biomarker sources [10]. EVs are lipid bilayer membrane vesicles containing bioactive contents (e.g., proteins, lipids and nucleic acids) [11]. Almost all cells are able to produce EVs, with their contents changing when the cell of origin changes due to (patho)physiology [12,13]. Due to this, EVs are often referred to as the “liquid biopsy”. The ability to study their (variable) contents makes them an interesting source for future biomarkers.

In this review, we first provide an overview of the performance of existing plasma biomarkers in CCS. Second, we review the existing evidence with regard to the additional value that EV biomarkers might have in diagnosing CCS. Last, despite an increasing number of publications regarding EVs as biomarker, the use of EVs in the cardiovascular field is not yet fully established. The use of EVs were recently incorporated into clinical practice in the cancer field of medicine [14,15,16]. We highlight several clinical aspects that need to be addressed in future studies to accelerate successful clinical implementation of EVs in the cardiovascular field.

## 2. Current Diagnostic Plasma Biomarkers in CCS

The use of biomarkers to detect CCS are studied extensively. Multiple promising markers were identified using a proteomics or metabolomics approach. However, new markers often fail when applied to an external and/or different population [17,18,19]. The focus of this review is on proteins and their function as biomarker, however, RNA, DNA or other cell particles in theory could also function as biomarkers.

### 2.1. Single Plasma Biomarker Approach

After the successful implementation of hs-cTn to diagnose ACS, identification of biomarkers with a similar accuracy for other coronary pathologies, such as CCS, received a lot attention. Many different markers for CCS were proposed, with the best known being natriuretic peptides, hs-cTn and C-reactive protein (CRP).

#### 2.1.1. Natriuretic Peptides

Natriuretic peptides, both B-type (BNP), and the N-terminal of the prohormone (NT-proBNP) are secreted as result of myocardial stretch [15]. Two studies investigated the diagnostic potential of natriuretic peptides in patients with stable angina who underwent CAG [20,21]. Weber et al. found NT-proBNP as an independent predictor for obstructive CCS in a small cohort study of 94 patients. They found an area under the curve (AUC) of 0.72 at a cutoff level of 214 pg/mL [20]. Additionally, a larger, comparable study performed in 781 patients found the same association but different cutoff points for men (85 pg/mL), with an AUC of 0.72, and women (165 pg/mL), with an AUC of 0.71 [21]. Both studies excluded patients with known heart failure or left ventricular ejection fraction of <60%. A meta-analysis performed in 2009 included 14 studies with a total of 2784 participants. They found a pooled sensitivity for the detection of stress-induced myocardial ischemia with 71% (NT-pro)BNP, however the pooled specificity was only 52% [22]. The performance of (NT-pro)BNP was consistent throughout different studies but remains limited compared to clinical models [23,24,25,26,27]. Jensen et al. showed an overview of five commonly used clinical risk scores and their performances in a large cohort of 5414 patients [28]. The AUCs of all clinical models varied between 0.68–0.72. Since most BNP studies also showed AUCs of ~0.70, the limited value of BNP on top of clinical models is not surprising. This was also seen when the performance of BNP (AUC 0.66) was compared with a clinical judgement score (AUC 0.66) [29].

#### 2.1.2. High-Sensitive Cardiac Troponin

Hs-cTn is well known for its role in diagnosing ACS, and, since it is a marker of cell damage caused by myocardial ischemia, it might also be helpful in diagnosing CCS. Higher levels of hs-cTn in patients without ACS were observed in patients that were older, had high systolic blood pressure, an increased left ventricular mass and/or renal impairment [30]. Hs-cTn was shown to be associated with the severity of CAD on CAG [31,32]. Moreover, a modest increase in AUC (0.79 to 0.80) to detect CCS in addition to a clinical judgement score was found [33]. However, this finding was not replicated in other large cohorts [29]. Tanglay et al. investigated the incremental value of a single hs-cTn measurement to rule out stress-induced myocardial ischemia and found an AUC to detect stress-induced ischemia with hs-cTn of 0.70 compared to an AUC of 0.69 from their clinical judgement model (*p* value = not significant) [34].

#### 2.1.3. C-Reactive Protein

CRP is an inflammatory marker, but also an acute-phase protein, and considered to be a nonspecific marker of inflammation [35]. Among all inflammatory biomarkers studied in CAD, CRP requires the most attention; unfortunately, the value of CRP to diagnose CCS appears to be limited [19,36]. The association between CRP and the extend of CAD was studied in a large cohort (>2500 participants) referred for CAG because of typical chest pain [37]. Only very modest correlation coefficients between CRP and CAD severity were found (r: 0.02–0.08). Another study investigating the diagnostic potential of CRP failed to show a statistically significant association between plasma CRP levels and obstructive CAD [38]. Large Mendelian randomization studies analyzing polymorphisms of the CRP gene also did not provide evidence of a causal relationship between CRP and CAD [39,40,41].

### 2.2. Multimarker Approach

After it was recognized that a single biomarker approach might not be able to improve the accuracy of clinical models to detect CCS, multimarker models were introduced. The idea behind a multimarker approach is the ability to combine different markers, all representing different pathophysiological pathways, thereby providing complementary information. Studies investigating a multimarker approach in diagnosing CCS are limited. One study investigated a dual-biomarker strategy to detect CCS [29], comparing the diagnostic accuracy of a clinical judgement score with BNP and hs-cTn. The addition of hs-cTn to the clinical judgement score significantly improved the diagnostic accuracy (AUC: 0.68 to 0.75), however, a dual marker strategy did not further improve the diagnostic accuracy.

Although multimarker models are studied in more detail regarding the prognosis of CAD patients, until now, the incremental value of multimarker models in future risk stratification was disappointing. Wang et al. studied 3532 patients from the Framingham Offspring Study and found that a high multimarker score was independently associated with both the outcome death as well as major adverse cardiovascular events (MACE) [42]. The multimarker score for death comprised CRP, NT-proBNP, homocysteine, plasma renin and urine albumin-to-creatinine ration. For MACE, two markers were selected: NT-proBNP and urine albumin-to-creatinine ratio. However, no significant differences in C-statistics were found when comparing a model with clinical predictors (death: 0.80, MACE: 0.76) with a multimarker model (death: 0.82, MACE: 0.77). Another study among >5000 patients without known cardiovascular disease (CVD) analyzed the predictive ability of both single and multimarker models on top of clinical predictors [43]. They analyzed two outcomes, namely, coronary events (selected markers were MR-proADM and NT-proBNP) and MACE (selected markers were CRP and NT-proBNP). Only a very modest increase in C-statistic (0.007 for MACE and 0.009 for coronary events) was found when using a multimarker model compared to a model with clinical predictors. Also, no significant reclassification of patients into higher or lower risk categories was found. Comparable results were found in studies with patients with manifest CVD [44,45]. Nevertheless, a multimarker approach could be the solution for a future CCS marker, but perhaps from another, relatively unexplored source, such as EVs.

## 3. EV Origin

Extracellular vesicles are characterized by a bilayer lipid membrane layer [11]. EVs were reported for the first time in 1946 by Chargaff and West [46], however, they were first recognized by Wolf in 1967 [10]. He observed EVs at that time as “platelet dust”. Following his endeavor, a lot of knowledge on EVs emerged. Almost all different cell types are able to produce and release EVs. EVs are found systemically and in basically all body fluids, including blood, urine, cerebrospinal fluid, milk, tears and saliva [47,48,49,50,51,52,53,54]. Characterization and classification of subpopulations have been subject of debate for the last years, with a consensus still not reached [55,56]. As a common feature, all subpopulations of EVs contain bioactive contents (lipids, proteins and nucleic acids). EV contents originate from the parent cell they are released from [57,58]. Once released into the extracellular space, parts of them can be identified to serve as cell-cell communicators.

### EV Subpopulations

**Figure 2:**
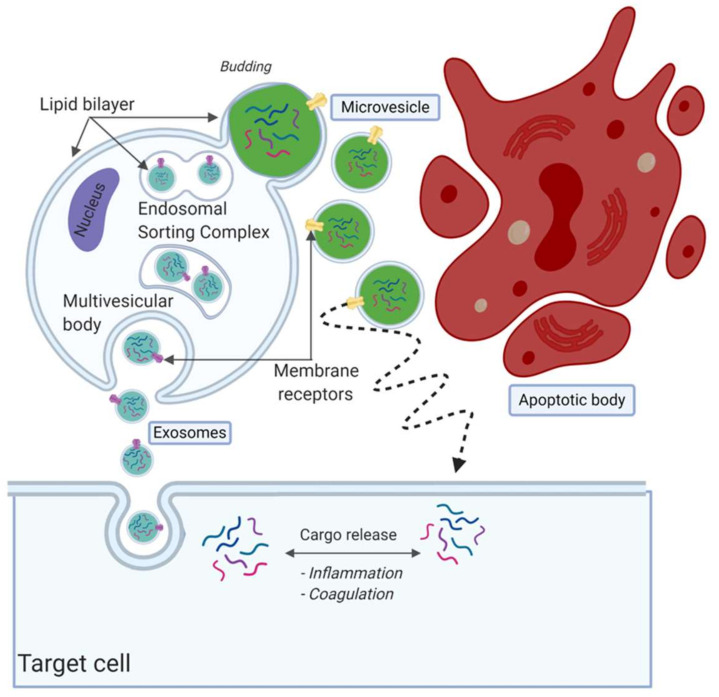
Overview of extracellular vesicle (EV) subpopulations and formation routes. EVs are often divided into three subpopulations, namely, exosomes, microvesicles and apoptotic bodies. Exosomes are considered the smallest population, released by fusion with the plasma membrane. Microvesicles are secreted by blubbing, as can be seen in green. Lastly, apoptotic bodies are fragments released from cells during apoptosis, considered to be the largest in size. Created with BioRender.com.

Although it is an ongoing debate regarding how to classify the EV subpopulations, EVs are often divided in three subtypes based on their size and formation route, namely, apoptotic bodies, microvesicles and exosomes [59,60,61] (Figure 2). There is no consensus on specific identifying protein markers to distinguish between the three subpopulations [62,63,64]. Exosomes are considered as the smallest particles in the EV family, with a size of 30–150 nm [65]. The release and formation of exosomes is via the endosomal sorting complex release transport (ESCRT) pathway [66]. They are formed as intraluminal vesicles and mature into multivesicular bodies (MVBs) [57]. MVBs fuse with the outer plasma membrane to be released within the extracellular space [67]. It was suggested that multiple subpopulations of exosomes exist, potentially providing additional information on their origin and role [65]. Microvesicles are EVs that form by outward budding, sometimes called blubbing, of the cell membrane. Their size is approximately between 100 nm and 1000 nm [59,60,61,68,69]. The last subpopulation of EVs are the apoptotic bodies, which are released after cell death. They are >1000 nm in size and relatively large compared to exosomes and microvesicles.

## 4. EVs as Diagnostic Biomarkers in Atherosclerosis

Atherosclerosis is considered to be the underlying syndrome of cardiovascular disease. EVs are considered to be key mediators in both the atherosclerotic plaque formation and its progression. EVs are thought to be involved in inflammation and thrombus formation and are therefore thought to carry useful information to serve as biomarkers [70,71]. Clinical risk factors associated with CAD are diabetes mellitus, hypertension, metabolic syndrome, hypercholesterolemia and smoking [72,73]. Several studies showed higher levels of circulation EVs in plasma to be associated with some of these risk factors [74,75], including diabetes [76,77], hypertension [78,79], hypercholesterolemia [80,81] and smoking [82,83,84]. Moreover, associations between EVs and subclinical atherosclerosis (diagnosed with ultrasound of the femoral artery, carotid artery or abdominal aorta) were also found [85,86]. Within CVD, EVs are the most studied as prognostic markers in CAD [87,88,89]. However, less is known about their diagnostic potential for CCS.

### 4.1. Extracellular Vesicle Count in CCS

One way to analyze plasma EV levels is to measure the number of circulating EVs, also described as count. There is an increasing number of publications on EVs in the cardiovascular field, however the subject of this review is the presumed role of EVs specifically in CCS. We focus specifically on their diagnostic potential in CCS patients. Table 1 provides a preselected overview of studies that investigated the role of EV counts of different subpopulations based on cellular origin with regard to CCS. Some of the studies are described below in more detail. Chironi et al. showed that the number of circulating leukocyte-derived EVs (LDEVs) were independently associated with subclinical atherosclerosis [85]. A study among 33 postmenopausal women undergoing coronary calcium scoring on coronary CT showed a positive association between the number of circulating EVs and both the Framingham risk score (FRS) as well as coronary calcium scores [86]. The effect of our circadian rhythm on the levels of circulating EVs was studied by a Scandinavian group in 30 patients, of which 10 had CCS and 20 patients were healthy controls [90]. They found a slight variation in total circulating EV count and the circadian rhythm, but no effect was seen for platelet-derived EVs.

CCS is characterized by stress-induced ischemia. Augustine et al. showed an increase in circulating EVs after dobutamine stress echocardiography, except for the patients with signs suggestive for stress-induced ischemia [91]. Sinning et al. [92] found a similar result of diminished EV release after stress-test imaging in patients with significant CCS, emphasizing a dynamic process of EV release. Several studies showed differences in the number of circulating (subpopulations of) EVs between patients with CCS and healthy controls, but also between patients with CCS and ACS [93,94,95,96,97,98]. These were however, mainly studies in small cohorts and were often cross-sectional. Mirachi et al. compared levels of two species of endothelial-derived EVs (EDEVs) (CD31+ and CD51+) between 84 patients with CAD (64 ACS and 20 CCS) and 42 healthy controls [93]. Levels of CD31+ EDEVs differed significantly between the ACS, CCS and controls, whereas CD51+ EDEVs only differed between CAD versus control, however no differences between ACS and CCS were observed. Additionally, this study also investigated levels of platelet-derived EVs (PDEVs), showing only elevated levels in patients with ACS. No differences were seen between CCS and ACS or CCS and controls [93]. Another study performed by Biasucci et al. compared levels of EDEVs, PDEVs and circulating EVs (cEVs) in 76 patients [97]. In this study population, 33 patients were diagnosed with CCS and 43 with ACS. All EV subpopulations were found in significantly higher levels in patients with ACS compared to patients with SA. They also investigated whether the levels changed over time, which was seen only for the total amount of circulating EVs [97]. There are contradicting results regarding circulating EV levels and the degree of luminal stenosis. Werner et al. showed a significant (adjusted) correlation between levels of circulating EVs and luminal stenosis [99], whereas two other studies did not [98,100]. Only a few studies investigated the diagnostic or prognostic properties of the number of circulating EVs in CCS patients. The largest study was performed by Nozaki et al. [88], showing in 378 CCS patients that endothelial-derived EVs were an independent predictor for MACE (hazard ratio (HR): 1.35 95%; confidence interval (CI): 1.09–1.65). Their prognostic model had an AUC of 0.73 and included the FRS as a clinical prediction rule and plasma biomarkers (CRP and BNP). After addition of the total count of endothelial-derived EVs, this increased to an AUC of 0.76. These findings were in line with other comparable studies showing the same results [87,89,101].

A different way to analyze subpopulations of EVs is to divide them based on density. This concept of EV separation was derived from a study showing a reduced amount of EVs in patients with familial hypercholesterolemia who underwent LDL apheresis [102]. EV subpopulations are still relatively unexplored and need to be studied in more detail to provide answers on the biological and pathophysiological functions [103]. They could, however, reflect different origins and a better signal-to-noise ratio, thereby providing additional information.

### 4.2. Extracellular Vesicle Content

When the (patho)physiological circumstances of a cell change, not only the number of EVs secreted by this cell changes, but also their content [11,104]. Compared with the count, data on the role of EV content in CCS is limited. Both EV nucleotides as well as protein content were described in CVD [104]. RNA quantification relies on the very sensitive and established technology of qPCR [105]. RNA has, however, the disadvantage of rapid degeneration by RNAse, which is present at high levels in blood. Further, mRNA levels often do not correlate with encoded proteins or reflect the ongoing biological process. Protein levels reflect much closer the ongoing process, and quantification is done by using immunoassays that are commonly used in clinical laboratories. Therefore, we think that proteins have the largest potential in diagnosing CCS [11,104].

One of the first studies that looked into the EV proteome was performed by Vélez et al. The main focus of the study was to explore whether they could study the proteome of EVs by comparing 10 ST-elevation myocardial infarction (STEMI) patients with 10 CCS patients [106]. They found 117 differentially regulated proteins between the two groups, indicating a potential source for protein markers. Another study compared EV-protein levels between STEMI patients and healthy controls [107]. Protein differences were analyzed with a proximity extension assay (Olink, CVD-II panel, N = 92) on the EV lysates and plasma. They identified three proteins (chyotripsin C, tyrosine-protein kinase (SRC) and C-C chemokine ligand 17) that showed differences in levels in EVs but not in plasma. Validation in another set of STEMI patients, CCS patients and healthy controls exposed CRS to be significantly associated with the degree of CAD. This finding was not found in plasma, indicating the additional diagnostic value of EVs.

**Figure 3:**
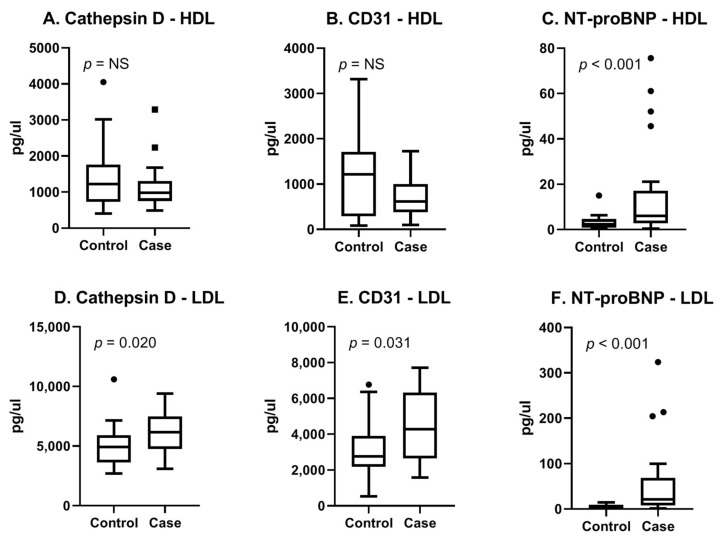
Boxplots of three selected proteins measured with MSD. Assessment of reproducibility of Olink results with a clinically available immunoassay. (**A**–**C**) HDL and (**D**–**F**) LDL indicate EV-subpopulations. Cases were 22 male patients with proven CCS and controls were 22 age- and risk-factor-matched patients who were symptomatic without CCS. Original assay units are pg/uL.

The myomarker study cohort consisted of consecutive patients presenting with stable chest pain at the outpatient clinic of the Meander Medical Centre in the Netherlands. Details on the study design and study population can be found in a previous publication [108]. For this study, a case control analysis of 44 men suspected of CCS was performed. Cases were defined as patients with stress-induced ischemia determined with MPI. Controls were matched based on age and general cardiovascular risk factors (Supplementary Table S1). In this cohort, we performed proteomics on EV subpopulations (rather than a total EV population) based on density since we hypothesized that this would provide a more detailed view of the cell condition. For this, we separated two subpopulations (called the HDL subpopulation and LDL subpopulation, respectively), as described in the study of Wang et al. [109]. We analyzed in the HDL- and LDL subpopulations using both the cardiometabolic panel as well as the cardiovascular III panel (Olink, Proteomics, Uppsala University Sweden). Each panel consisted of 92 proteins known for their associations with CVD. We identified the three most promising proteins (Cathepsin D, CD31 and NT-proBNP) based on literature, their diagnostic properties, and the availability of antibodies (Supplementary Table S2). Using the Meso Scale Discovery (MSD, Rockville, MD, USA) immunoassay, we confirmed our findings. Figure 3A–C shows boxplots of the MSD results for the selected three proteins in the EV-HDL subpopulation. The results for the LDL subpopulation are summarized in Figure 3D–F. It can be appreciated that in the EV-LDL subpopulation, protein levels of Cathepsin D, CD31 and NT-proBNP were significantly higher in cases compared with controls. In the HDL subpopulation, only NT-proBNP-protein levels were found to be significantly different between cases and controls. Our results therefore show the potential of using the Olink technology for the enrichment of EV proteins in EV subpopulations, followed by confirmation in an established immunoassay.

For EV-based diagnosis of CCS, not many data exist. A recent study investigated whether a selected group of EV-proteins were associated with CCS [108]. EV-Serpin C1, EV-CD14, EV-Serpin G1, EV-Serpin F2 and EV-Cystatin C (mostly in the HDL-subpopulation) were shown to be independently associated with the presence of stress-induced ischemia. The prognostic value of EV-protein content in a large CVD cohort was for described for the first time by Kanhai et al. [110]. They found EV-Cystatin C, EV-Serpin F2 and EV-CD14 protein levels to be independently associated with future cardiovascular events. A different study found an independent association between the extent of CVD and the levels of EV-CD14 [111]. Several other studies investigated the role of EV content in ACS, heart failure, unstable angina and manifest CVD [112,113,114].

## 5. Clinical Aspects of CCS Diagnosis Using (EV) Blood Tests

The population suspected of CCS is very heterogenous, ranging from patients presenting with clear symptoms and obstructive CAD to patients with nonspecific chest pain without obstructive CAD and everything in between. The current ongoing search towards a biomarker for more accurate detection of CCS is being developed to apply to all patients suspected for CCS but, considering the heterogeneity in this population, this should raise questions. A study performed by Ouellete et al. found clear differences in the clinical profile of patients with respectively normal, near normal, nonobstructive CAD and obstructive CAD [115]. These differences in clinical profiles between the groups seem obvious but are important in the development of a future biomarker. A more patient-tailored search for a future biomarker focusing on specific subgroups seems reasonable. Moreover, considering the fact that EV content enable us to look at cellular level, one could imagine the EV content of a patient with a known history of CAD is not comparable to a patient with new-onset disease.

An important subject within this heterogeneity that merits consideration is sex. Although sex differences are established and acknowledged regarding clinical symptoms and pathophysiology, the exact underlying mechanisms are barely understood [116]. Evolving knowledge supports the differences in pathophysiology, diagnostic test performance and also prognosis [117,118,119]. Women tend to have less obstructive CAD and more often a preserved ejection fraction, yet higher mortality rates and more extensive myocardial ischemia [118]. Women often present with more complex signs and symptoms. It was suggested that this is due to a more complex and multifactorial pathophysiological process compared to men [119]. A large study investigating biomarkers within CVD showed a difference in protein profile between men and women of almost 85% [120]. Research performed in EVs also showed differences in the associations of both EV count as well as content with clinical outcomes stratified on clinical factors [103,108,112].

These data and hypotheses raise the question whether future studies on biomarkers should focus on predefined subgroups of patients rather than the entire “suspected CCS” group. It emphasizes the need to incorporate clinical aspects associated with CCS into future studies with EVs. From our point of view, the most important clinical aspects that merit attention are sex, age and the cardiovascular status of a patient. This cardiovascular status refers to whether or not a patient is already known with atherosclerotic disease or if a patient previously received (invasive) treatment. Until now, EV studies in CCS did not have enough power to perform reliable subanalyses to reveal different associations within this heterogenous group. It might be possible that we need to develop different biomarkers, or cut-offs, within the entire group of patients suspected of CCS.

## 6. Future Perspectives

Despite great efforts of the internation society of extracellular vesicles (ISEV) to standardize EV research and improve reproducibility, it remains difficult to compare results between studies [56]. This is mainly because studies still use numerous different techniques for isolation and quantification [121]. There are various protocols for sample preparation, processing and centrifugation, which are known to cause different results [122]. Currently, flow cytometry is the most used method to quantify EVs (see also Table 1). This method is standardized and accepted for the identification and detection of different cell types, however, is most reliable for particles >200 nm [123]. Considering the fact that most EVs are around 100–120 nm in the blood on average, it is questionable whether this is the best method to count circulating EVs. Also, it does not enable measuring EV contents besides proteins stained on the EV membrane.

### 6.1. Automation

One reason why the use of EVs in clinical practice is hampered is the inability to use high-throughput isolation techniques [122]. Currently, ultracentrifugation is often used to isolate EVs from whole plasma, however, this is time-consuming, labor intensive and requires many manual steps [107]. Before clinical implementation of EVs is considered, large confirmatory trials are needed [104]. Considering the current time effort, costs and the amount of precious clinical blood used for the isolation and quantification of EVs, this is a disillusion. Future studies should therefore focus on development of an automated method for EV isolation, purification and downstream analysis [124], ideally using very small sample volumes to improve chances for clinical implementation.

### 6.2. Internal Standard

The use of a reliable internal standard would also increase the chances for clinical implementation. As rightly opposed by Loyer et al., despite efforts to identify specific subpopulations of EVs with specific membrane markers, very few studies report the purity of their obtained subpopulations [13]. Improvement can be obtained with an internal standard for the number of EVs per milliliter plasma in a sample. For this, a housekeeping protein present in all EVs (e.g., beta-actin) might be a way of developing such a standard. Alongside this, an internal control to visualize the loss of EVs during isolation is needed. Labeled synthetic beads or liposomes might be used for this. Already, these two standards could improve reproducibility and accuracy of EV count and content and measurements in precious clinical samples.

### 6.3. Future Directions

Future studies should focus on clinical applicability by developing internal standards and introduce automation and standardization of EV isolation and quantification [56]. Larger cohorts are warranted in order to derive valid clinical prediction models that enable the added value of EV contents as biomarkers to be shown, particularly when taking the heterogeneity within CCS patients into account.

Since EV protein content are based on established immunoassays and are increasingly showing merit in the diagnosis and prognosis of CVD, including the potential for automation and standardization, we expect this to prevail in this field in the next few years.

Although technical challenges still have to be resolved, we anticipate that EVs will be used as a reliable source for research into the diagnosis and prognosis of CCS in the next few years. This could potentially contribute to more personalized medicine and a more efficient use of our healthcare system.

## Figures and Tables

**Table 1:** Overview of preselected publications on extracellular vesicle count in chronic coronary syndrome patients, including details on subpopulations.

Study Characteristics				Extracellular Vesicles			Study Findings	
Name, Year	N (%Male)	Design	Population	Subpopulation	Identifier	Method		Ref.
Jayachandran, 2008	33 (0)	Cross	Newly postmenopausal women undergoing CT CAC	cEVs	AnnexinV+	FC	Higher in women with high CAC, associated with FRS	[86]
				PDEVs	CD61+/CD42a+	FC	Higher in women with high CAC, associated with FRS	
				GDEVs	CD11b+	FC	NS, NA	
				MDEVs	CD14+	FC	NS, NA	
				EDEVs	CD62e+/AnnexinV+	FC	Higher in women with high CAC, associated with FRS	
Christersson, 2015	30 (53)	CC	CCS pts (CAG+) vs. healthy controls	cEVs	AnnexinV+	FC	Slight circadian variation	[90]
				PDEVs	CD41+/CD62+	FC	NA	
				EDEVs	CD144+/CD14+	FC	Sign. higher levels in the morning	
Augustine, 2014	119 (45)	Co	Consecutive pts undergoing DSE	cEVs	AnnexinV+	FC	Sign. rise and fall after DSE in patients without ischemia	[91]
				PDEVs	CD31+/CD41+	FC	Sign. rise and fall after DSE in patients without ischemia	
				EryDEVs	CD235a+	FC	Sign. rise and fall after DSE in patients without ischemia	
				EDEVs	CD31+/CD41−, CD62e+, CD106+	FC	Sign. rise and fall after DSE in patients without ischemia	
				LDEVs	APC+	FC	NS	
				GDEVs	CD66b+	FC	NS	
				MDEVs	CD14+	FC	NS	
Sinning, 2016	80 (71)	Co	Consecutive pts undergoing DSE and CAG	EDEVs	AnnexinV+/CD31+	FC	Decrease after DSE in patients with ischemia	[92]
				MDEVs	AnnexinV+/CD14+	FC	Decrease after DSE in patients with ischemia	
				PDEVs	AnnexinV+/CD31+/CD42b+	FC	NS	
Tan, 2009	89 (49)	CC	CCS pts referred for CAG	PDEVs	CD61+/CD42b+	FC	Sign higher in CCS, NA with severity of luminal stenosis	[98]
Stęogonekpień, 2012	30 (73)	CC	CCS pts vs. ACS vs. control pts no CCS criteria defined	PDEVs	CD42+	FC	CCS vs. control NS. ACS vs. CCS Sign	[96]
				LDEVs	CD45+	FC	CCS vs. control NS. ACS vs. CCS Sign	
				MDEVs	CD14+	FC	CCS vs. control NS. ACS vs. CCS Sign	
				EDEVs	CD31+, CD34+, CD51+/CD61+	FC	CCS vs. control NS. ACS vs. CCS Sign	
				TFDEVs	CD142+	FC	CCS vs. control NS. ACS vs. CCS Sign	
Biasucci, 2012	76 (74)	Obs	CCS pts referred for CAG vs. ACS	cEVs	CD31+/AnnexinV+	FC	CCS vs. ACS Sign. Sign decrease over time	[97]
				PDEVs	CD31+/CD42b+	FC	CCS vs. ACS Sign. NS decrease over time	
				EDEVs	CD31+/CD42b-	FC	CCS vs. ACS Sign. NS decrease over time	
Mizrachi, 2003	108 (NR)	CC	CCS pts vs. ACS vs. controls	EDEVs	CD31+, CD51+	FC	CCS vs. control Sign	[93]
				PDEVs	CD42+	FC	NS (any subgroup)	
Mallat, 2000	52 (69)	NR	CCS (CAG+) vs. ACS vs. Non cardiac controls	cEVs	AnnexinV+	PA	CCS vs. Control Sign. ACS vs. CCS Sign	[95]
				NR	CD3+	NR	NS	
				NR	CD11a+	NR	NS	
				NR	CD31+	NR	NS	
				NR	CD146+	NR	CCS vs. Control Sign. ACS vs. CCS Sign	
				NR	GP-Ib+	NR	NS	
Werner, 2005	50 (68)	Co	CCS (CAG+), acetylcholine	EDEVs	CD31+/AnnexinV+	FC	Sign. (adjusted) correlation with luminal stenosis	[99]
Song, 2015	73 (45)	Co	CCS pts undergoing CAG	EDEVs	CD144+/AnnexinV+	FC	Intermediate lesion vs. no lesion Sign. Not correlated with degree of stenosis	[100]
Nozaki, 2009	378 (61)	Long	CCS pts (CAG+ or >2riskfactors)	EDEVs	CD144+	FC	Independently associated with MACE HR1.35 (95% CI 1.09–1.65)	[88]
Sinning, 2011	200 (70)	Long	CCS pts (CAG+)	EDEVs	CD31+/AnnexinV+	FC	Independently associated with MACE HR 2.3 (95% CI 1.3–3.9)	[87]
Koga, 2005	234 (57)	CC	CCS pts (CAG+) +DM vs. control	EDEVs	CD144+/CD42b−	FC	CCS + DM vs. control Sign. Predictor of presence CCS (OR 4.1 95% CI 2.20–7.70)	[89]
Hu, 2014	33 (48)	CC	CCS pts (CAG+) vs. control	EDEVs	CD31+/CD42b−	FC	NS	[101]
					CD62e+	FC	CCS vs. control Sign. Diagnostic accuracy AUC: 0.80	
				PDEVs	CD41+	FC	NR	

Design: Cross = Cross-sectional; Co = Cohort; Long = Longitudinal; CC = Case Control. Population: CAC = Coronary Artery Calcium; CCS = Chronic coronary syndrome; CAG = Coronary angiography (+ indicates proven with this modality); DM = Diabetes Mellitus; ACS = Acute Coronary Syndrome; pts = patients; DSE = Dobutamine Stress Echocardiography. Subpopulation: cEV = Circulating EV; PDEVs Platelet-derived EVs; EDEVs = Endothelial-derived EVs; GDEVs = Granolycyt-derived EVs; MDEVs = Monocyte-derived EVs; EryDEVs = Erythrocyte-derived EVs; LDEV = Leukocyte-derived EVs; TFDEVs = TF+-derived EVs. Method: FC = Flowcytometry; PA = Protrombinase Assay. Study findings: FRS = Framingham Risk Score; Sign = Significant *p* value < 0.05. NR = Not reported; NS = Not significant; NA = Not associated.

## Supplementary Materials

Supplementary materials can be found at https://www.mdpi.com/1422-0067/21/23/9128/s1.
