# Supplementary material for: Extracellular Vesicles in Diagnosing Chronic Coronary Syndromes the Bumpy Road to Clinical Implementation
Source: Int J Mol Sci. 2020 Nov 30;21(23):9128. doi: 10.3390/ijms21239128 (PMC7729611; doi:10.3390/ijms21239128)
Supplement: Supplementary file 1 [file ijms-21-09128-s001.pdf]

Supplemental table S1. Baseline characteristics of the Myomarker study cohort

	Control	Case	P value
n	22	22	
Age	63.32 (10.27)	67.86 (16.23)	0.273
BMI	29.79 (4.20)	27.97 (4.31)	0.174
**Risk factors**			
Smoking	5 (22.7)	2 (9.1)	0.410
Diabetes	6 (27.3)	6 (27.3)	1.000
Hypertension	12 (54.5)	14 (63.6)	0.759
Hypercholesterolemia	12 (54.5)	12 (54.5)	1.000
Family history of CAD	8 (36.4)	10 (45.5)	0.759
**Medical history**			
Cardiovascular disease	16 (72.7)	19 (86.4)	0.455
Coronary artery disease	9 (40.9)	12 (54.5)	0.546
Coronary revascularization	8 (36.4)	10 (45.5)	0.759
**Medication**			
Anti hypertensive drugs	15 (68.2)	18 (81.8)	0.486
Lipid lowering drugs	13 (59.1)	14 (63.6)	1.000
Anticoagulans	2 (9.1)	5 (22.7)	0.410
Antiplatelet	11 (50.0)	13 (59.1)	0.762

Values are shown as mean (SD) or number with corresponding frequency. Case is defined as stress-induced ischemia objectified with myocardial perfusion imaging. CAD = Coronary Artery Disease.

Supplemental table S2. Diagnostic performance selected proteins

Biomarker	Controls	Cases	p value	AUC	Sens(%)	Spec(%)	PPV(%)	NPV(%)
LDL-Cathepsin D	0.44 [0.38-0.51]	0.55 [0.45-0.64]	0.007	0.74	68.2	81.8	78.9	72
LDL-CD31	1.50 [1.28-1.90]	2.05 [1.64-2.81]	0.004	0.75	68.2	72.7	71.4	69.6
LDL-NT-proBNP	1.22 [1.04-1.41]	1.98 [1.35-2.85]	<0.001	0.81	68.2	95.5	93.8	75
HDL-Cathepsin D	0.24 [0.23-0.29]	0.26 [0.23-0.30]	0.573	0.55	68.2	54.5	60	63.2
HDL-CD31	0.80 [0.71-0.85]	0.92 [0.81-1.15]	0.015	0.72	68.2	72.7	71.4	69.6
HDL-NT-proBNP	0.99 [0.86-1.22]	1.30 [1.16-1.66]	0.001	0.78	86.4	63.6	70.4	82.4

Sens = Sensitivity, Spec = Specificity, PPV = positive predictive value, NPV = negative predictive value.
Case = symptomatic patient with proven CCS, control = symptomatic patients without CCS.
